# CERN-MEDICIS: A Review Since Commissioning in 2017

**DOI:** 10.3389/fmed.2021.693682

**Published:** 2021-07-15

**Authors:** Charlotte Duchemin, Joao P. Ramos, Thierry Stora, Essraa Ahmed, Elodie Aubert, Nadia Audouin, Ermanno Barbero, Vincent Barozier, Ana-Paula Bernardes, Philippe Bertreix, Aurore Boscher, Frank Bruchertseifer, Richard Catherall, Eric Chevallay, Pinelopi Christodoulou, Katerina Chrysalidis, Thomas E. Cocolios, Jeremie Comte, Bernard Crepieux, Matthieu Deschamps, Kristof Dockx, Alexandre Dorsival, Valentin N. Fedosseev, Pascal Fernier, Robert Formento-Cavaier, Safouane El Idrissi, Peter Ivanov, Vadim M. Gadelshin, Simone Gilardoni, Jean-Louis Grenard, Ferid Haddad, Reinhard Heinke, Benjamin Juif, Umair Khalid, Moazam Khan, Ulli Köster, Laura Lambert, G. Lilli, Giacomo Lunghi, Bruce A. Marsh, Yisel Martinez Palenzuela, Renata Martins, Stefano Marzari, Nabil Menaa, Nathalie Michel, Maxime Munos, Fabio Pozzi, Francesco Riccardi, Julien Riegert, Nicolas Riggaz, Jean-Yves Rinchet, Sebastian Rothe, Ben Russell, Christelle Saury, Thomas Schneider, Simon Stegemann, Zeynep Talip, Christian Theis, Julien Thiboud, Nicholas P. van der Meulen, Miranda van Stenis, Heinz Vincke, Joachim Vollaire, Nhat-Tan Vuong, Benjamin Webster, Klaus Wendt, Shane G. Wilkins, the CERN-MEDICIS collaboration

**Affiliations:** ^1^Organisation Européenne pour la Recherche Nucléaire (CERN), Geneva, Switzerland; ^2^Katholieke Universiteit (KU) Leuven, Institute for Nuclear and Radiation Physics, Leuven, Belgium; ^3^Groupement d'Intérêt Public ARRONAX, Nantes, France; ^4^European Commission, Joint Research Centre, Nuclear Safety and Security, Karlsruhe, Germany; ^5^National Physical Laboratory, Teddington, United Kingdom; ^6^Johannes Gutenberg University, Mainz, Germany; ^7^Pakistan Institute of Nuclear Science and Technology, Islamabad, Pakistan; ^8^Institut Laue Langevin, Grenoble, France; ^9^Paul Scherrer Institute, Villigen, Switzerland

**Keywords:** CERN, MEDICIS, medical, radionuclides, mass separation

## Abstract

The CERN-MEDICIS (MEDical Isotopes Collected from ISolde) facility has delivered its first radioactive ion beam at CERN (Switzerland) in December 2017 to support the research and development in nuclear medicine using non-conventional radionuclides. Since then, fourteen institutes, including CERN, have joined the collaboration to drive the scientific program of this unique installation and evaluate the needs of the community to improve the research in imaging, diagnostics, radiation therapy and personalized medicine. The facility has been built as an extension of the ISOLDE (Isotope Separator On Line DEvice) facility at CERN. Handling of open radioisotope sources is made possible thanks to its Radiological Controlled Area and laboratory. Targets are being irradiated by the 1.4 GeV proton beam delivered by the CERN Proton Synchrotron Booster (PSB) on a station placed between the High Resolution Separator (HRS) ISOLDE target station and its beam dump. Irradiated target materials are also received from external institutes to undergo mass separation at CERN-MEDICIS. All targets are handled via a remote handling system and exploited on a dedicated isotope separator beamline. To allow for the release and collection of a specific radionuclide of medical interest, each target is heated to temperatures of up to 2,300°C. The created ions are extracted and accelerated to an energy up to 60 kV, and the beam steered through an off-line sector field magnet mass separator. This is followed by the extraction of the radionuclide of interest through mass separation and its subsequent implantation into a collection foil. In addition, the MELISSA (MEDICIS Laser Ion Source Setup At CERN) laser laboratory, in service since April 2019, helps to increase the separation efficiency and the selectivity. After collection, the implanted radionuclides are dispatched to the biomedical research centers, participating in the CERN-MEDICIS collaboration, for Research & Development in imaging or treatment. Since its commissioning, the CERN-MEDICIS facility has provided its partner institutes with non-conventional medical radionuclides such as Tb-149, Tb-152, Tb-155, Sm-153, Tm-165, Tm-167, Er-169, Yb-175, and Ac-225 with a high specific activity. This article provides a review of the achievements and milestones of CERN-MEDICIS since it has produced its first radioactive isotope in December 2017, with a special focus on its most recent operation in 2020.

## Introduction

**Figure 1:**
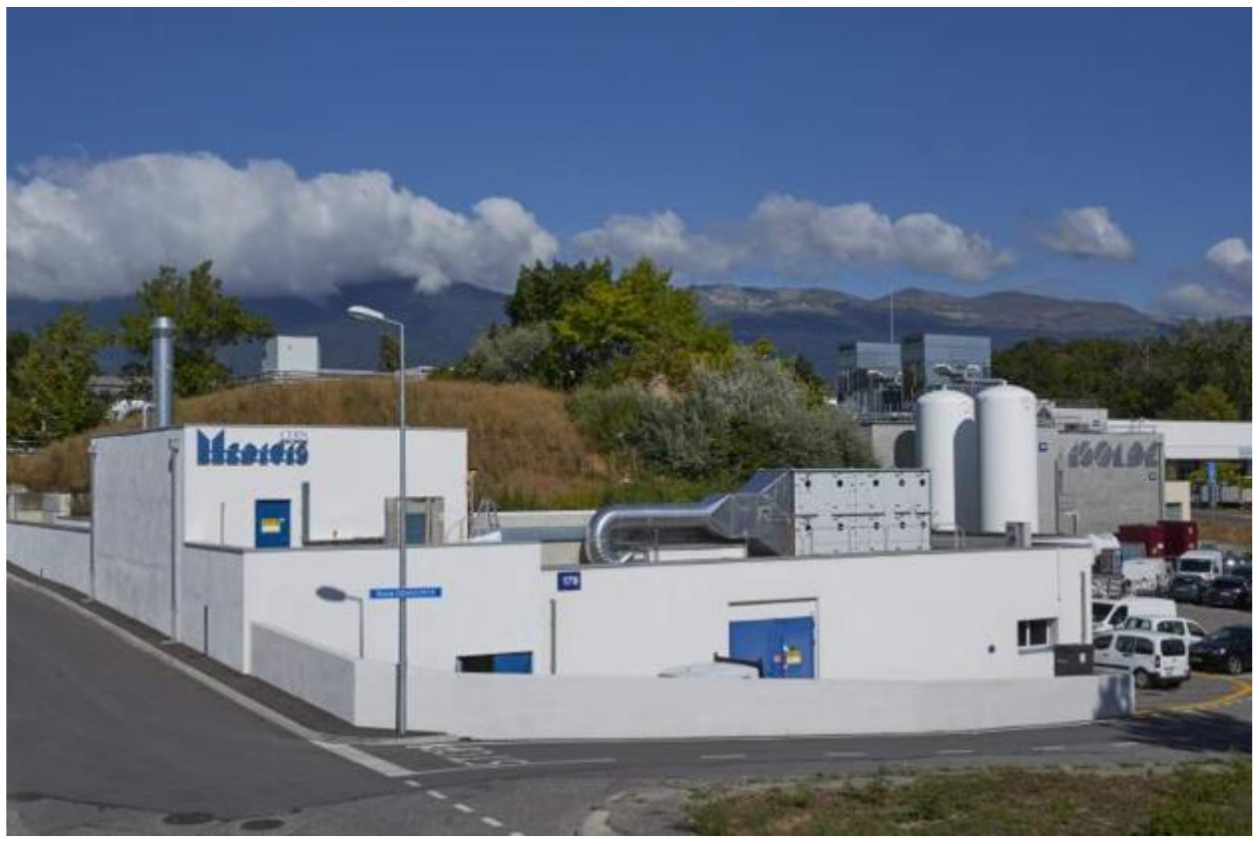
The CERN-MEDICIS building completed in 2017.

Since the publication of the first official use of radionuclides administered to a patient to treat cancer in the 1930s, huge progress has been made. Several radionuclides are currently widely available as radiopharmaceuticals and mainly used to either diagnose or treat cancer. Research in nuclear medicine is ongoing with a growing interest in personalized treatment and diagnosis, the so-called theranostics approach. Adapting the treatment to each patient's pathology requires to have a large panel of approved radiopharmaceuticals available in order to give access to novel and diverse treatment modalities. The big advantage of personalized and targeted treatment is that individual pathologies can be taken into account and the destruction of the surrounding healthy tissue can be minimized by careful selection of the adequate radionuclide. In order to obtain such radiopharmaceuticals, one needs to produce a specific radionuclide with the highest isotopic and chemical purities within a standardized workflow and in sufficient quantities. These radionuclides can be created via the irradiation of a stable target material in particle accelerators or in nuclear reactors. However, additional processes are usually needed to reach the purity level necessary for the preclinical experiments and clinical trials. Depending on the radionuclide of interest, such purification can be attained either by means of chemical separation or by combining mass and chemical separation. Based on the strong expertise in mass separation of radioisotopes existing for more than 50 years at CERN's Isotope Separator On-Line DEvice facility (ISOLDE) (1), a project dedicated to medical applications has been initiated by CERN in 2010. The idea behind this new and unique facility is to produce non-conventional radionuclides having the required properties for both imaging and treatment as well as to expand the range of radionuclides available for the medical research in hospitals and in research centers across Europe. The facility has been funded with contributions from the CERN Knowledge Transfer Fund, private foundations and partner institutes, as well as benefitting from a European Commission Marie Skłodowska-Curie training grant titled MEDICIS-Promed. After the ground-breaking in September 2013 this new facility (see Figure 1), baptized MEDICIS (MEDical Isotopes Collected from ISolde), entered its commissioning phase in autumn 2017 (2). In Europe, a number of facilities producing radioactive beams by online isotope mass separation (ISOL) are currently operating such as ISOLDE at CERN, ALTO at IJC-Lab and SPIRAL-1 at GANIL, while ISAC at TRIUMF in Canada is also exploiting ISOL rare-isotope beams. While these facilities can technically produce isotopes for medical applications, their research activities are focused on fundamental and applied studies in nuclear physics with pure exotic radioactive beams through mass separation. Currently, CERN-MEDICIS is the only European facility which dedicates its full program to the production and delivery of medical isotopes for research in radiopharmaceutical science, operating in batch isotope mass separation mode. CERN-MEDICIS is also at the heart of a new European project called PRISMAP which is a consortium of 23 institutes in order to translate the emerging radionuclides into medical diagnosis and treatment, in which isotope mass separation plays an important role to achieve appropriate specific activities or radionuclidic purities. In the future, the SPES facility in Italy and the ISOL@MYRRHA facility in Belgium also aims to produce pure exotic radioactive beams and medical isotopes.

## The MEDICIS Collaboration and Its Research Projects: From Its Beginnings to the Present

CERN-MEDICIS produced its first radionuclides for medical research after an off-line mass separation on the 12th of December 2017. Tb-155 was the first radionuclide collected at MEDICIS, of the four terbium radioisotopes that are highly promising for cancer diagnosis and treatment. After this successful and promising commissioning phase, CERN-MEDICIS formally became a collaboration the year after, with the signature of the Memorandum of Understanding and the first collaboration board meeting held at CERN. The members of the collaboration (3) are experts in medical radionuclide production, nuclear medicine, radiochemistry and nuclear research. They hail from research institutes, hospitals and universities: GIP ARRONAX (France), CHUV (Switzerland), EANM (Europe), FABIS (Spain), HUG (Switzerland), ILL (France), IST (Portugal), JGU Mainz (Germany), JRC Karlsruhe (Germany), KU Leuven (Belgium), NPL (UK), PSI (Switzerland), PAEC (Pakistan), RTU-LU (Latvia). PAEC and RTU-LU officially joined the MEDICIS collaboration in 2021 (see Table 1). Biomedical projects are regularly submitted to the collaboration board, which evaluates the needs of the community as well as the technical feasibility and provides recommendations. In that way, the CERN-MEDICIS scientific program and list of radionuclides are defined. Since 2018, 31 projects have been submitted to the collaboration board in the biannual collaboration meetings that have already taken place (see Table 1). Through the list of approved projects (3), one can see strong interest in lanthanides and particularly terbium radioisotopes including the alpha emitter Tb-149 (4), the positron emitter Tb-152 (5) and the gamma and Auger emitter Tb-155 (6). The medical and scientific community also identified some scandium radioisotopes, such as Sc-44 for Positron Emission Tomography (PET) (7) and Sc-47 for use in both therapy and Single Photon Emission Computed Tomography (SPECT) (8). Cu-67 is a radionuclide being proposed among the projects that would be well-suited for theranostic applications (9). CERN-MEDICIS focuses also on the delivery of mass-separated Sm-153, Tm-167, Er-169, Yb-175, Hg-191/Pt-191 and the alpha emitter Ac-225. From the first year of operation in 2018 to the end of 2020, MEDICIS has provided nine different external research institutes or hospitals with 41 batches of high specific-activity radionuclides. This has been done within the framework of 12 approved projects. Since 2017, production and mass separated isotopes at CERN-MEDICIS support ongoing research programs by providing high purity products which are not accessible in cyclotrons or reactors without mass separation. Even though some of the above-mentioned radionuclides can be efficiently produced in reactors or cyclotrons, they are produced with isotopic impurities that can only be removed by going through a mass separation process. For example, Ac-227 will be a co-product of Ac-225 and some isotopes such as Tb-153, Tb-154, Tb-156 will be generated as contaminants of Tb-155. High Specific Activity (HSA) radionuclides from neutron activated targets can also be provided, such as HSA Er-169 which is otherwise not achievable.

**Table 1:** MEDICIS collaboration boards, number of institutes taking part in the MEDICIS collaboration, number of submitted projects and list of radionuclides of interest.

**Board number**	**Date**	**Number of institutes in the collaboration**	**Number of projects**	**Radionuclide(s) of interest**
1	21/02/2018	12	13	C-11, Sc-43, Sc-44, Sc-47, Cu-67, Xe-131m, Xe-133m, Tb-149, Tb-152, Tb-155, Er-169
2	03/10/2018		3	Sc-44, Sc-47, Tb-149, Tb-155
3	20/03/2019		7	Fe-52, Fe-59, Tb-149, Tb-152, Tb-155, Tm-167, Er-169, Yb-175, Pt-191, Pt-193m, Pt-195m
4	18/09/2019		1	Ac-225, Ac-227
5	20/02/2020		1	Sm-153
6	17/09/2020		2	Cu-64, Ac-225
7	11/03/2021	14	4	Ba-128/Cs-128, Ce-134/La-134, Tb-149, Tb-152, Ac-225

## MEDICIS' Modes of Operation for Radionuclide Production

One of the main features of CERN-MEDICIS is that it can profit from several irradiation possibilities to produce its isotopes before proceeding to the off-line mass separation of the radionuclide of medical interest (10). The facility has the opportunity to irradiate targets at CERN in the ISOLDE primary area. Every target unit is compatible with both, the ISOLDE and MEDICIS facilities, and is composed of an aluminum water-cooled vacuum vessel. The latter encloses a tubular tantalum oven inside of which a target material, ready for irradiation, is placed. This oven is connected to an ion source via a transfer line [more details can be found in (11)]. The MEDICIS target is installed for irradiation behind one of the ISOLDE's target station (HRS) and before the beam dump via an automatic rail conveyor system (RCS). The MEDICIS target can be:

- directly irradiated by the 1.4 GeV proton beam delivered by the CERN Proton Synchrotron Booster (12);
- indirectly irradiated by the fraction of the primary proton beam (>65%) which did not interact with the HRS target, as well as by its secondary particle showers [see more details in (10)] – so-called parasitic mode.

**Figure 2:**
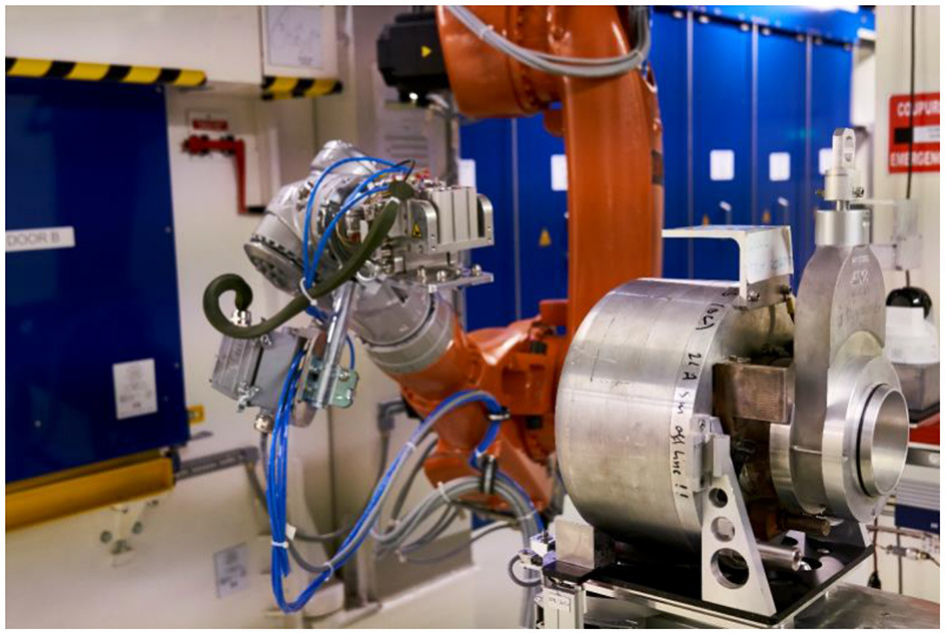
The MEDICIS robot about to transfer a target from the RCS to the target station.

Once the MEDICIS target has been irradiated, it is transported back to a decay point via the same RCS. From this point onward a dedicated robot, from the KUKA^®^ company, handles the target (see Figure 2) and is used to safely connect the target to the MEDICIS target station to subsequently start with the collection of the radionuclide of interest. It should be noted that with this mode of operation and since the full target unit is subjected to the proton and secondary particle fluences, not only the target material located inside the target oven is activated but the full unit is. In 2017 and 2018, 11 targets were irradiated and used for mass separation at CERN-MEDICIS, with some of them irradiated up to five times.

However, since the start-up of the Large Hadron Collider (LHC), accelerator operation at CERN is intermitted by extended upgrade and maintenance periods called Long Shutdowns (LS). During these periods the full accelerator chain is stopped and no protons can be delivered to the various CERN experiments. The first LS took place from February 2013 to mid-2014, followed by the second from January 2019 to mid-2021. CERN-MEDICIS is one of the very few facilities at CERN which was still operating during the second Long Shutdown (LS2). During the years 2019 and 2020, CERN-MEDICIS performed off-line mass separation of medically important radionuclides from materials irradiated at external partner institutes. This operation mode is being exploited since the first successful feasibility test carried out in 2018 with the mass separation and the collection of 18 MBq of Er-169 from naturally abundant Er-168, irradiated in the reactor of ILL in Grenoble (France) (13, 14). Each externally irradiated material to be mass separated is shipped to CERN-MEDICIS and it arrives either in sealed quartz vials or inside a dedicated sample holder, developed at CERN in 2019. It is a tight fit made of a tantalum cylinder with an inner part rhenium foil lining, coming with its plexiglass protection. The latter has been designed to prevent any contamination of the transport container and to guarantee an easy, rapid and safe transfer of the externally irradiated sample into the empty oven of the target unit. It was made to avoid any risk of dispersion and contamination as well as to limit the radiation exposure of the operator. All the externally irradiated materials imported to CERN-MEDICIS in 2020 were received inside this new piece of equipment and no contamination incident has been reported to date. In the case of reception of sealed quartz vials, the decontamination, opening and transfer into the target's empty oven were performed at CERN-MEDICIS using a dedicated automatic transfer system. Once the target unit is loaded with the radioactive material, it is handed over to the robot which couples it onto the MEDICIS target station in view of the mass separation and collection. It should be noted that in the case of externally irradiated material and in contrast to the mode of operation that involves the irradiation with protons at CERN in the ISOLDE target area, there is no activation of the target unit itself.

In 2019 and 2020, CERN-MEDICIS received and used 34 externally irradiated target materials. These radioactive samples were provided by the GIP ARRONAX in Nantes (France), ILL in Grenoble (France), JRC in Karlsruhe (Germany), PSI in Villigen (Switzerland) and SCK CEN in Mol (Belgium).

**Figure 3:**
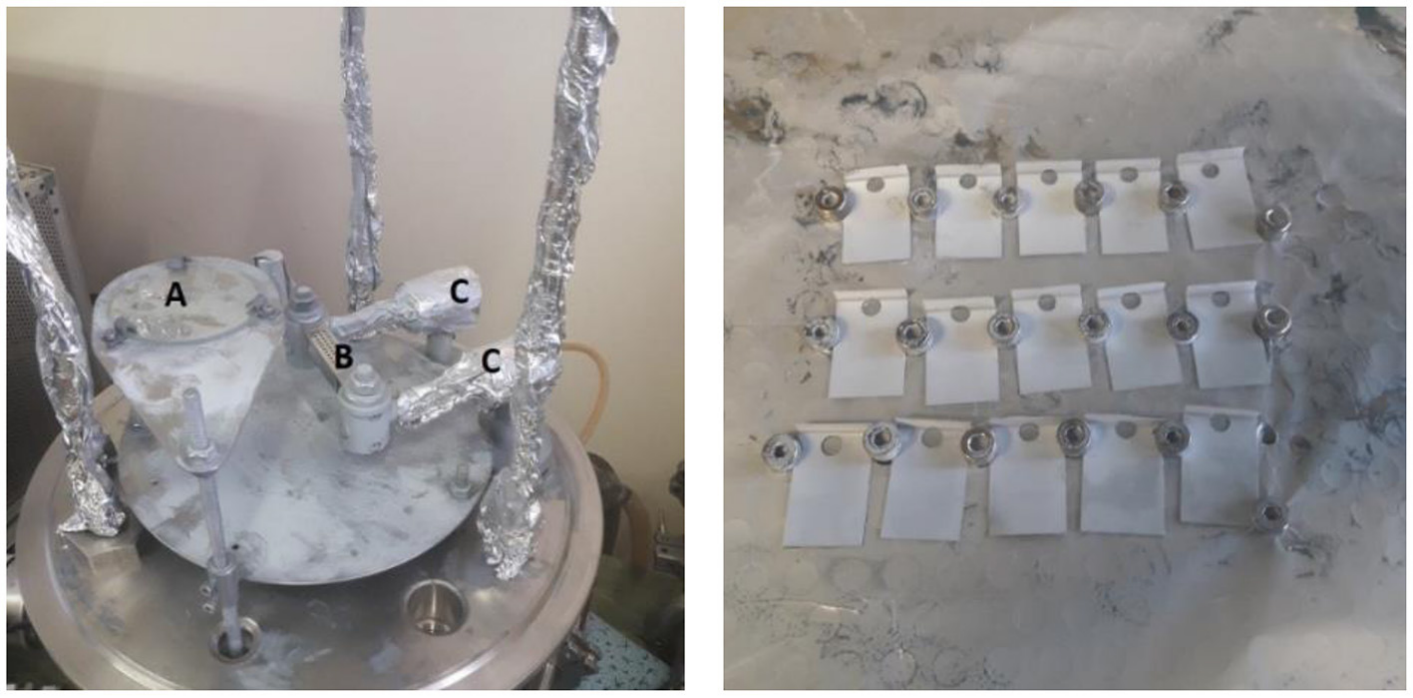
PVD set-up with zinc filled molybdenum boat (picture on the left) with **(A)** the shutter, **(B)** the Molybdenum crucible with Zinc granulates and **(C)** the High Voltage lines – Zinc-coated gold foils (picture on the right).

Regardless of the irradiation conditions and modes utilized, each target unit, once coupled to the target station by the robot, is heated up to very high temperatures to allow for the diffusion and effusion of the isotopes of interest. Even though the optimal temperature differs for each target material and radionuclide to be mass separated, these temperatures often reach more than 2,000°C. The isotopes pass through an ion source, where they are ionized and subsequently accelerated to be sent through a mass separator (dipole magnet) as Radioactive Ion Beams (RIBs). More information regarding the MEDICIS beam line can be found in (15). Furthermore, the MELISSA laser laboratory (16, 17) helps to increase the separation efficiency and the selectivity. The extracted radionuclides are usually implanted onto thin zinc-coated gold foils. The foils are prepared under high vacuum using 99.995% pure zinc granulate thermally heated in a molybdenum boat and evaporated onto the 0.25 mm thick gold substrates (Figure 3). With the assistance of a build-in INFICON thickness sensor the layer thickness is determined to 500 nm in this Physical Vapor Deposition (PVD) process. Preparation and cleaning of the gold plates (surface roughening and ultrasonic cleaning) is crucial for the zinc adherence and layer uniformity.

After the implantation, the foils are safely retrieved from the collection chamber and transported to a shielded fume hood by using a shielded trolley. This is followed by their shipment to one of CERN-MEDICIS' partner institutes.

## MEDICIS' Operation From December 2017 to December 2020: A Review

### 2017: The First Radioactive Beam

**Figure 4:**
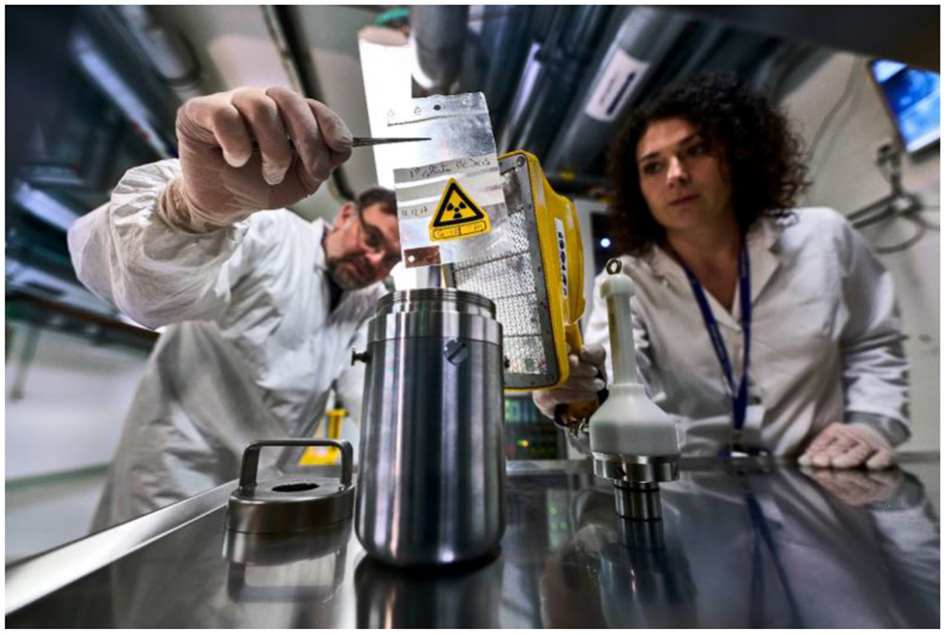
Dose rate measurement on the first collection of Tb-155 at CERN-MEDICIS.

The 10th of November 2017 marked the beginning of CERN-MEDICIS' operation with the start of hardware commissioning (power converters) and the polarization at 30 kV of the ion source platform for secondary ion beam generation. It was followed by the extraction of the first stable isotope beam 5 days later. The first target, containing 250 g of tantalum rolls as material inside the target oven, was irradiated for 24 h on the 5th of December 2017. One month later, the commissioning of the facility was completed with the first collection of Tb-155 (18) (see Figure 4). One Tb-155 sample was also shipped to the IST Lisboa (Portugal).

### 2018: A Full Year of Operation With the CERN Proton Beam

The year 2018 began with a technical stop of several months and stable beam tests, before CERN's proton beams were available again. In May 2018, CERN-MEDICIS was ready again to operate with irradiated targets and launch its first operation year. Beamtime was devoted to nine different approved projects through the year. It included several developments within the MEDICIS-Promed European Commission Marie Sklodowska-Curie innovative training program with, as examples, the successful separation of Er-169 from externally irradiated Er-168 (13, 14) as feasibility tests in preparation for LS2, as well as promising C-11 diffusion studies (19). In total, 5.5 × 10^19^ protons have been directed to the ISOLDE HRS target station in 2018, among which 44% could be exploited for the MEDICIS program (i.e., 2.4 × 10^19^ protons). It includes 6.0E18 protons (11%) that have been directly sent to the MEDICIS irradiation point by deflecting the proton beam below the upstream ISOLDE target while the latter was being set up for physics runs. This direct irradiation mode allows for considerably increasing the activity, which can be produced in the MEDICIS targets. Based on FLUKA Monte Carlo (20, 21) simulations (CERN version 4.1), a study performed on the radionuclide production in such a target being either directly or indirectly irradiated (see scenarios presented in section MEDICIS' Modes of Operation for Radionuclide Production) has been carried out (22). It focused on the activity of several radionuclides of interest for CERN-MEDICIS produced after different irradiation times ranging from 1 h to several days, in combination with 1 h of cooling time. For the indirect irradiation scenario, a UCx target has been placed upstream of the MEDICIS target. As a result, the gain in the activity produced using the direct irradiation mode ranges on average between 12 and 15 for Ac-225, Sc and Tb radioisotopes (see Table 2).

**Table 2:** Predicted activity gains from a direct irradiation in comparison to the indirect mode.

**Target material**	**Titanium**		**Tantalum**		**ThO**_****2****_	
Radionuclide	Sc-44	Sc-47	Tb-149	Tb-152	Tb-155	Ac-225
Activity ratio $\frac{\text{Direct}}{\text{Indirect}}$	15	13	14	13	12	15

It should be noted that the high activity levels after the retrieval of the target units did not allow for quantitative activity measurements by gamma-ray spectroscopy. Dose rate measurements of the full target units have been carried out which confirm an expected notable gain in activation levels after direct irradiation. For example, a comparison of the previously described scenario yielded a ratio of 10 in the case of Ta target units. However, one should keep in mind that this value should be understood as an indication as it is based on measurements of the full target unit and not of a specific radionuclide.

Five radionuclides of medical interest were collected in 2018: Er-169, Tb-149, Tb-152, Tb-155 and Tm-165, a generator of the Auger electron emitter Er-165. The collected activities ranged from 1 to 137 MBq with separation efficiencies up to 1.6% (10). Two research institutes, the Centre Hospitalier Universitaire Vaudois (CHUV) in Lausanne (Switzerland) and the National Physical Laboratory (NPL) in Teddington (UK), respectively, received batches of Tb-149 and Tb-155. Within this framework, MEDICIS successfully showed its capability to collect and deliver the short half-life radionuclide Tb-149 (T_1/2_ = 4.1 h) with a delay between the end of the target irradiation and the shipment departure of less than two half-lives. A total of 1.7 GBq of activity was handled in 2018, including the activity coming from isobars/impurities. Out of this value, 235 MBq could be exploited for medical applications. Twelve target units, including prototypes, were used for the CERN-MEDICIS program in 2018. Some of these targets were irradiated up to five times. This mode of operation significantly reduces the amount of generated radioactive waste and the costs. Including machine development runs, 220 h were devoted to the collection of radionuclides in 2018. CERN-MEDICIS profited from 20 irradiations slots and could proceed with 15 collection campaigns (see summary in Table 3). Although typically performed on zinc-coated gold foils, preliminary tests have been performed with the implantation of radionuclides in KNO_3_ salt layer deposited on thin aluminum foils. The latter has been done within preliminary radiochemistry developments performed at CERN-MEDICIS and in view of simplifying the post-implantation radiochemistry process by recovery of the implanted activity in buffered aqueous solutions.

**Table 3:** Summary of the collections performed at CERN-MEDICIS in 2018, 2019 and 2020.

**Year**	**Irradiation modes**	**Medical isotopes**	**Collected activity (MBq)**	**Maximum collection efficiency^a^ (%)**	**Number of batches delivered**	**Number of projects concerned**	**Number of new targets**
2018	CERN PSB & external irradiations	C-11, Tb-149, Tb-152, Tb-155, Tm-165, Er-169	235	1.6	4	2	12
2019	External irradiations	Tb-155, Er-169, Yb-175, Pt-195m	870	6.0	15	5	8
2020	External irradiations	Sm-153, Tb-155, Tm-167, Ac-225	540	22.5 (53% separated^b^)	16	5	3

a *Calculated as the ratio between the total activity measured on the collection foils at the end of the collection and the activity present inside the target container at start of the collection*.

b *An efficiency of 53% has been measured by the on-line γ-spectrometer but due to sputtering effects, part of the activity has been lost on the foils' support and inside the collection chamber*.

After a second collaboration board meeting on the 3rd of October 2018 (see Table 1), additional irradiation slots on the MEDICIS irradiation point were approved to extend the so-called ISOLDE nuclear physics winter program. Long-lived radionuclides were further extracted at the beginning of CERN's shutdown period, with Be-7 and radium monofluoride molecule beams, producing one of the ISOLDE physics result highlights (23).

After the last proton beam delivered at CERN on the 12th of November 2018, CERN-MEDICIS entered its technical stop period for maintenance and upgrade.

### 2019: A Year Without Protons at CERN Compensated by the Use of Externally Irradiated Target Materials

**Figure 5:**
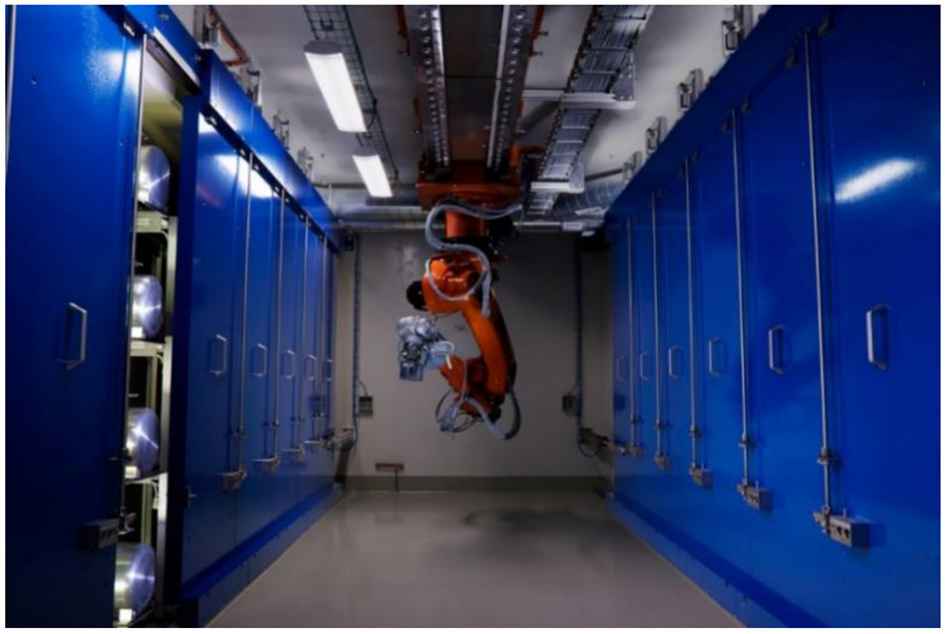
The shielded storage shelves and the robot.

CERN-MEDICIS' second year of operation started with a 6 months technical stop dedicated to maintenance and upgrade, followed by a commissioning phase. Notably, in February the MEDICIS target storage shelves became fully operational (see Figure 5).

**Figure 6:**
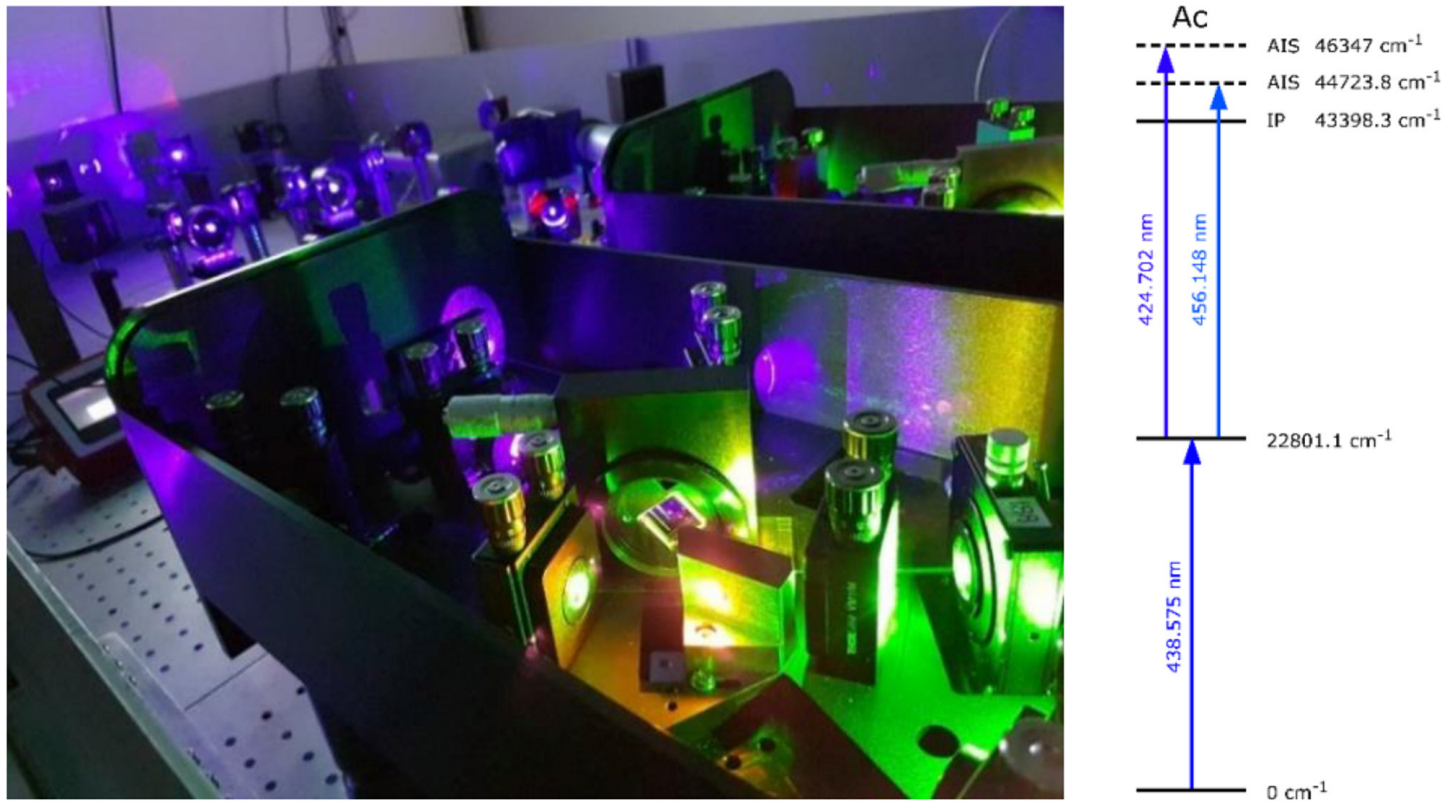
MELISSA laser set-up operational since April 2019 (picture on the left) and laser resonance ionization scheme for actinium (picture on the right).

It was followed by the successful commissioning of the shielded fume hood in March into which the collection foils can be safely removed from their support, and where radiochemistry developments can be performed. Another milestone was reached in 2019 when the MELISSA laboratory (16, 17) became fully operational in April. The installed laser setup consisted of two Z-cavity Ti:sapphire lasers of the Mainz University/CERN design pumped by two 10 kHz pulsed Nd:YAG lasers InnoLas Nanio 532-18-Y (see Figure 6). Using intra-cavity frequency, doubling blue beams required for two-step resonant ionization of rare-earth elements could be generated by this setup. The operating principle of MELISSA setup is identical to that of the ISOLDE resonance ionization laser ion source (RILIS) system (24). Since then, all collections performed at CERN-MEDICIS have profited from the added value and selectivity provided by MELISSA. The laser resonance ionization scheme for actinium (25, 26) is given as an example in Figure 6. A regularly updated compilation of laser schemes applied at RILIS systems can be found at http://riliselements.web.cern.ch/.

In June 2019, the CERN-MEDICIS target station was back in operation after the complete replacement of a defective extraction electrode on the MEDICIS beamline. The first collection of radionuclides started on the 2nd of July. The externally irradiated enriched Er-168 containing Er_2_O_3_ target was imported from ILL (France), from which 79 MBq of Er-169 was collected for shipment to PSI (Switzerland). CERN-MEDICIS operated in 2019 with target materials irradiated either at the nuclear research reactor of ILL or at the GIP ARRONAX cyclotron (France) until the end of the year.

Er-169, Yb-175 and Pt-195m have been produced in the reactor of ILL by neutron capture on enriched Er-168, Yb-174, and Pt-194, respectively. Due to the high quantity of stable isotopes present in the samples after neutron irradiation, mass separation allows to significantly increase the specific activity of the radionuclide of interest. An activity of 92 MBq of Pt-195m was shipped to the Hopitaux Universitaires de Genève (HUG) in Switzerland for preliminary tests prior to future mass separation. Throughout the year 2019, a total of 350 MBq of mass separated Er-169 was provided to PSI, together with 520 MBq of mass separated Yb-175 for radiochemical separation, quality control and proof-of-concept preclinical experiments (27).

ARRONAX provided its first sample containing Tb-155 at the beginning of August 2019, to be mass separated. It was produced by irradiating natural gadolinium foils by the 30 MeV proton beam on target delivered by its cyclotron (28). The sample was shipped to CERN-MEDICIS to separate the Tb-155 in mass from the stable gadolinium target atoms as well as from the other produced terbium isotopes. Three additional externally irradiated samples were provided by ARRONAX throughout the year 2019. Given the difficulties in extracting terbium isotopes from the irradiated gadolinium material, several weeks of stable beam tests have been dedicated to the operation optimization and laser scheme developments. In addition, post-irradiation radiochemistry had been performed at ARRONAX in order to reduce the proportion of gadolinium atoms in the sample and increase the mass separation efficiency. Two batches of mass separated Tb-155 were delivered to the National Physical Laboratory in the UK and to KU Leuven/SCK CEN in Belgium for radiochemical studies, detector calibration and isotope qualification.

The year 2019 can be summarized as 16 collection campaigns carried out within 922 h of operation (not including the weeks of operation devoted to stable beam tests). A total of 870 MBq of mass separated activity were delivered to the institutes with the addition of 92 MBq of Pt-195m. Four institutes and five different approved research projects could profit from these radionuclides. Eight target units were used throughout the year, with some of them used up to three times. Moreover, collection efficiencies up to 6% (for Yb-175) could be achieved which represents a significant improvement in comparison with the operation year 2018 (see Table 3). Last but not least CERN-MEDICIS welcomed 1400 visitors during CERN's Open Days on the 14th and 15th of September 2019.

### 2020: Record Separation Efficiencies Achieved With Externally Irradiated Target Materials

**Figure 7:**
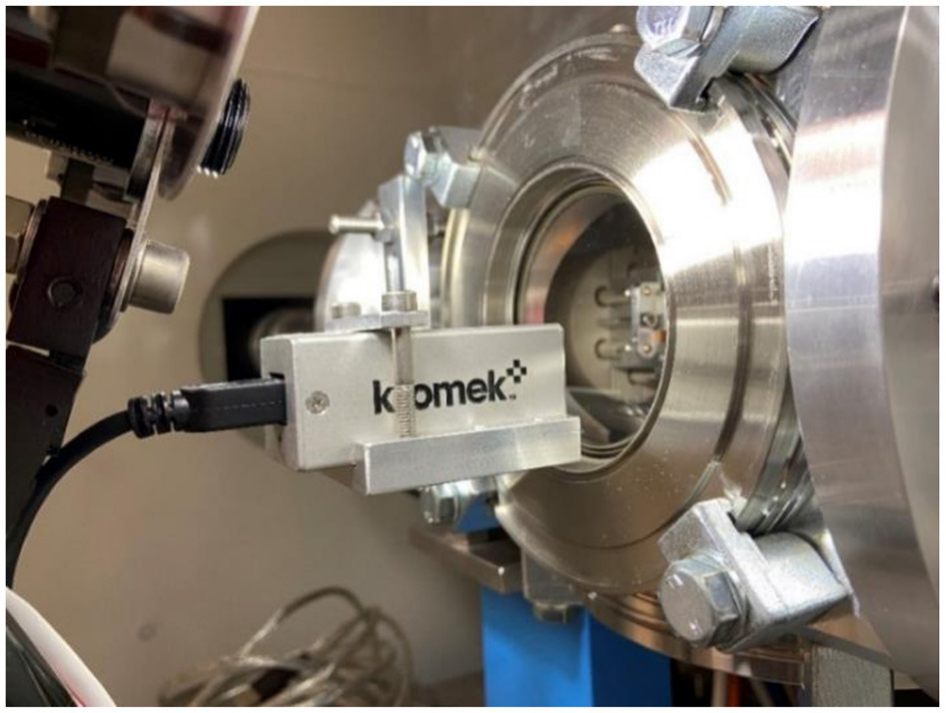
CZT γ-ray detector installed in front of the window of the collection chamber and performing the on-line measurement of the activity being implanted on the foils.

From January to March 2020 CERN-MEDICIS entered into a new technical stop for maintenance and upgrade. It included, among other works, the delicate replacement of a defective extraction electrode (a highly contaminated part) and the installation of a new gas injection system compatible with the use of chloride gas in view of producing molecular beams in the near future. Another improvement of the facility during the first semester 2020 was the integration of a compact Cadmium Zinc Telluride (CZT) γ-detector from Kromek^®^ for the on-line monitoring of the collected activity being implanted onto the collection foils (see Figure 7) (29). The activity being accumulated during a collection can thus be monitored together with the radionuclidic purity of the beam impinging on the foil. Monitoring the implantation rate has helped in the daily operation and allowed for a notable increase in the separation efficiency, as shown in the following.

During the 5th collaboration board meeting, a new project regarding the mass separation of Sm-153 from externally irradiated targets of Sm-152 was proposed (see Table 1) (3). The project was approved by the board members with high priority for 2020.

The technical stop ended at the beginning of March, followed by its commissioning. It included software and stable beam tests until the 16th of March 2020. From that day onwards all CERN installations suspended their activities due to the Covid-19 sanitary crisis. In total, CERN-MEDICIS' operation was stopped for 10 weeks. During that time the official CERN-MEDICIS website has been created as a portal for public outreach as well as exchange of information of the collaboration (3).

The operation of the facility restarted on the 25th of May, with the successful conditioning of the newly installed electrode, at voltages up to 65 kV. In parallel, an additional laser, provided by Mainz University, has been received and installed in MELISSA (see section 2019: A Year Without Protons at CERN Compensated by the Use of Externally Irradiated Target Materials). It is a grating-tunable Ti:sapphire cavity that considerably aids the scheme developments. Motorized etalon mounts and a laser beam stabilization system were installed to improve the long-term stability of the laser ion source. A beam imaging system was implemented to enable remote monitoring and diagnosis of the setup. These upgrades, combined with the additional laser system, allowed for improving the performance and reliability of the laser ion source during radionuclide collections as well as for increasing the number of elements for which the ion source can operate. In preparation of the near future mass separation of Sm-153, Tb-155 and Tm-167, the CERN-MEDICIS facility has dedicated several weeks of stable beam tests in synergy with MELISSA. From the use of an evaporated solution of Tb-159 (in 5% HNO_3_) deposited on a rhenium foil, 3% of terbium separation efficiency has been achieved with target temperatures above 2,200°C. Tests performed with stable samarium have shown a separation efficiency of up to 31% with an optimal operation temperature found around 1,700°C. This temperature has subsequently been confirmed during the radioactive samarium collections by monitoring the optimum implantation rate with the online CZT detector. In addition, thulium separation efficiency measurements were performed. A solution of natural thulium was deposited and evaporated on natural Er_2_O_3_, in order to better reproduce the operation conditions with the irradiated material. Efficiencies ranging from 65% (pure thulium) to 60% (thulium with erbium oxide surplus) were achieved and dedicated systematic measurements on the influence of the presence of contaminants on the laser ionization efficiency were performed by using different elements and ion source materials [more details can be found in (30)].

During the last week of June 2020 and after a successful, intensive and conclusive period of commissioning and stable beam production, the CERN-MEDICIS facility received the authorization to operate again with radioactive beams. The facility received its first externally irradiated target material of the year on 26th of June 2020. Radioactive ion beams have been produced at the end of June 2020 until the 9th of December 2020. The scientific program of the year focused on the mass separation, collection and delivery of four radionuclides of medical interest. It included the mass separation of Sm-153 (t_1/2_ = 46.5 h), which is a β^−^ emitter having the advantage of a γ-emission at 103 keV that is detectable by external imaging devices. Sm-153 was initially produced from enriched Sm-152 targets and neutron capture reactions at the SCK CEN BR2 reactor (31). Additional batches of mass-separated Tb-155 were provided by CERN-MEDICIS to two partner institutes. This radionuclide had been initially produced at ARRONAX and shipped to CERN-MEDICIS for further processing. Moreover, the therapeutic radionuclide Tm-167 was produced from natural erbium targets irradiated by 22.8 MeV protons, at the Paul Scherrer Institute (30). Two samples of Ac-225 were provided to CERN-MEDICIS by JRC Karlsruhe in order to perform preliminary studies on separation efficiency and optimal operation temperatures within the framework of a project proposal approved at the 4th MEDICIS collaboration board. The study of the mass separation of this radionuclide was performed one time with Ac-225 being only deposited and dried on a rhenium foil and a second time with Ac-225 being deposited on a sample of thorium oxide as potential future irradiation target material.

The two extractions of Ac-225, with Ac-225 alone and Ac-225 deposited on a sample of ThO_2_, led to separation efficiencies of 12.5 and 9.8%, respectively. Both were performed using surface and laser ionization. In the first scenario 1,900°C was found to be the optimal temperature, whereas temperatures above 2,300°C were necessary to extract the Ac-225 from ThO_2_. These results represent important foundations for the operation in 2021 and provide input to the mass separation of Ac-225 from ThO_2_ targets irradiated by the 1.4 GeV proton beam delivered by the CERN PS Booster. From the eight Sm-153 collection campaigns, the mass separated activities were ranging from 20 to 130 MBq. Depending on the initial activity present inside the target container at the start of the collection efficiencies of up to 12.7% were encountered. Separation efficiencies ranging between 1 and 6% were achieved during the four Tb-155 collections performed that year, to produce batches of mass separated Tb-155 with activities up to 20 MBq. By considering the activity given by the CZT γ-spectrometer which is monitoring the activity being implanted on the foils inside the collection chamber (see Figure 7), a maximum separation efficiency of 53% has been reached at CERN-MEDICIS that year, for the mass separation of Tm-167. However, during the three Tm-167 collection campaigns, sputtering effects of the implantation layer led to a loss of the activity on the frame of the foils' support as well as inside the collection chamber. From the activities measured after removal of the foils, a maximum efficiency value of 22.5% was observed (see Table 3). This figure is computed from the total activity measured on the collection foils during one collection campaign as a function of the activity which was present inside the target at the start of the collection on each foil (ranging from 75 to 120 MBq). The Tm-167 optimal operation temperature window was determined using progressing heating steps and found to be between 1,900 and 2,200°C. A detailed description is given in (30).

In 2020 a total of 17 collections have been performed with 16 batches shipped to four European partner institutes (PSI, SCK CEN/KU Leuven, CHUV and NPL). This corresponds to a total of 720 h of collection time. Only three new targets have been built for operation in 2020. Among these targets, one has been used 8 times and is still considered to be re-used in 2021 since no obvious sign of failure or decreased efficiency could be observed. In addition, three targets have been reutilized from 2019. Five approved projects could profit from high purity radionuclides delivered by CERN-MEDICIS in 2020 with a total of 530 MBq collected and shipped to our partners.

The radiochemistry activities have also progressed, starting from 2018. In 2020 the separation of the implanted isotopes from the zinc layer could take place at CERN-MEDICIS itself. Based on an ion-exchange chromatography, a method has been developed for the separation of the lanthanides collected in 2020 and has been tested for low activity levels, below 1 LA [Limite d'Autorisation according to the Swiss regulations (32)]. The parameters have been optimized specifically for three radiolanthanides, namely samarium, terbium and thulium. An automated system is also being developed to separate higher radioactivity levels. Irradiated metallic Pt-194 targets were converted into PtCl_2_ at the partner institute PINSTECH (Islamabad, Pakistan) for the MED-022 project and will be used either directly or for Pt separation tests. In addition, the treatment of concentrated liquid radioactive acidic waste was performed in order to transform it into easily disposable solid waste.

## MEDICIS in 2021 and Beyond

Since its commissioning in December 2017, CERN-MEDICIS has shown its capability to deliver non-conventional radionuclides to its partner institutes with a gradual and continuous improvement of its capabilities. Despite the global public health crisis, the year 2020 brought important technical, operational and scientific results, partly reflected in other manuscripts of this topical issue (15, 27, 30, 31). It has been a successful year for the MEDICIS facility as well as for the new European Medical Isotope program—PRISMAP—which has been selected for funding (33). PRISMAP, backed by a consortium of 23 institutes, was approved for funding by the Research Infrastructures program INFRA-2-2020 of Horizon 2020 of the European Commission, in which isotope mass separation has been identified as an important step in the production of radiopharmaceuticals. PRISMAP aims to federate European key stakeholders for the translation of emerging radionuclides into medical diagnosis and treatment. It has been initiated in May 2021, with mass separation and the CERN-MEDICIS facility at the center of the project.

The operation of the facility is stopped between January and May 2021 due to the construction of a new laboratory for the safe research and development of actinide nanomaterials as new target materials, which is being built as an annex to the CERN-MEDICIS laboratory. CERN-MEDICIS will continue providing its external partners with high specific activity radionuclides from June 2021 onward. Targets irradiated at CERN as well as externally irradiated materials will be exploited. The irradiation possibilities are foreseen to be extended thanks to a second irradiation station installed behind the second ISOLDE General Purpose Separator (GPS) target station. The results collected from the three past years of operation, together with the approved scientific program, have been used to set priorities on options for the upgrade of the facility. This notably includes studies to adapt the implantation layer to avoid sputtering, modification of the collection chamber for ion beam rasterizing and the possibility to collect multiple isotope beams in parallel. While the LHC injector upgrade (LIU) is coming to completion, proton beams from the PSB using the new Linac 4 injector will be available for the next 4 years. The next CERN Long Shutdown (LS3) will take place from the end of 2024 onward during which operation with external sources provided by the partner institutes, and produced in cyclotrons or reactors, will again become possible.

Within the CERN-MEDICIS collaboration and the PRISMAP European project, the list of isotopes will continue to be extended according to the needs of the community. By gaining experience during every year of operation, progressively larger activities will be produced and delivered to the research institute. With the aim to achieve a sustainable production scheme, which is among the goals of PRISMAP, further evolution could take place. This should be seen in the context of CERN's upgrade plans such as proton energy increases to 2 GeV or higher beam intensities that could further extend the present reach of the facility.

## Data Availability Statement

The original contributions presented in the study are included in the article/supplementary material, further inquiries can be directed to the corresponding author/s.

## Ethics Statement

Written informed consent was obtained from the individual(s) for the publication of any potentially identifiable images or data included in this article.

## Author Contributions

TSt: project leader. CD and JPR: project coordination. CD: initial and final draft manuscript. All authors manuscript review and contribution to MEDICIS operation since 2017. CD and TSt: final approval of the version to be submitted. All authors contributed to the article and approved the submitted version.

## Conflict of Interest

The authors declare that the research was conducted in the absence of any commercial or financial relationships that could be construed as a potential conflict of interest.
